# Advances in Experimentation and Numerical Modeling of Aluminum and Copper Ultrasonic Welding

**DOI:** 10.3390/mi16030263

**Published:** 2025-02-26

**Authors:** Zhe Li, Shiying Wu, Huan Li

**Affiliations:** 1 School of Electromechanical, Guangzhou Railway Polytechnic, Guangzhou 511300, China; lizhe@gtxy.edu.cn; 2 School of Mechanical Engineering, Yangtze University, Jingzhou 434000, China

**Keywords:** ultrasonic welding, research progress, dissimilar joints, microstructure, mechanical property, simulation analysis

## Abstract

Ultrasonic welding is characterized by its energy-saving and environmentally friendly nature. Compared to conventional molten welding technology, the intermetallic compounds formed by diffusion during ultrasonic welding are thinner, and material deformation is reduced. This process has become a primary welding technique for assembling lithium batteries in electric vehicles. Aluminum and copper ultrasonic welding has increasingly gained attention as a research hotspot. The research on aluminum and copper ultrasonic welding primarily focuses on the interfacial microstructure evolution, mechanical performance during the welding process, and numerical simulations to investigate macro- and micro-scale physical phenomena. Given the aluminum and copper multi-layer structures used in lithium battery packaging, numerous studies have been conducted on aluminum and copper multi-layer ultrasonic welding. For Al/Cu joints, advancements in understanding the microstructure evolution, joint performance, and finite element modeling of the welding process have been systematically reviewed and summarized. Moreover, significant progress has been made in molecular dynamics simulations of Al/Cu ultrasonic welding and hybrid welding techniques based on Al/Cu ultrasonic welding. Finally, several new research directions for Al/Cu ultrasonic welding and joining have been proposed to guide further in-depth studies.

## 1. Introduction

Aluminum and copper dissimilar welded joints are widely used in the assembly of power lithium batteries and electrical systems in electric vehicles due to the excellent electrical and thermal conductivities of aluminum and copper [1,2,3,4]. However, owing to the significant differences in physical properties between aluminum and copper, welding Al/Cu joints using traditional arc welding methods is challenging [5,6,7,8]. Nevertheless, due to the great difference in physical properties between aluminum and copper, it is easy for defects such as non-fusion and cracks to occur during traditional fusion welding. In recent years, the primary welding technologies for joining lithium battery pack tabs have included resistance spot welding, laser welding, and ultrasonic welding (USW). In resistance spot welding of Al/Cu joints, resistance heat flux occurs around the electrode rather than at the Al/Cu interface, which requires high welding energy input [9]. When laser welding aluminum and copper, a thick intermetallic compound is typically formed at the welding interface, making it difficult to control [10,11].

As a solid-state welding technology, ultrasonic welding is characterized by its energy efficiency and environmental friendliness [12,13,14,15]. The intermetallic compound layer generated during ultrasonic welding is thinner and more uniformly distributed. In addition, compared to conventional fusion welding techniques for Al/Cu joints, ultrasonic welding does not require water cooling or gas protection. It is also not sensitive to surface oxide films and does not require surface cleaning or additional welding materials. Currently, this technology has gained widespread attention because of its significant advantages in improving the weld quality of Al/Cu joints. As a primary welding technology for battery assembly, Al/Cu ultrasonic welding has become a research hotspot.

There are numerous research directions for Al/Cu ultrasonic welding, and many studies have been published on this topic. However, very few literature reviews on the ultrasonic welding of Al/Cu joints are available [16,17,18]. Additionally, advancements in new technologies, such as molecular dynamics simulation and multi-layer Al/Cu ultrasonic welding, have not been comprehensively summarized in existing reviews. Therefore, this review consolidates the research status of the ultrasonic welding process, finite element simulation, and nano-scale molecular dynamics simulation for Al/Cu joints. Finally, guidelines and valuable knowledge for the integration of aluminum and copper USW in lithium battery packs are provided as a foundation for future research directions in these fields.

### 1.1. The Principle of Ultrasonic Welding

**Figure 1:**
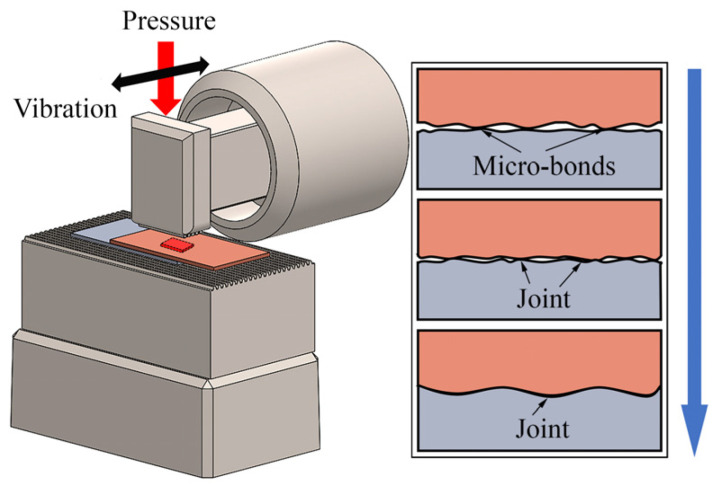
The principle of USW.

Ultrasonic welding converts electrical energy into mechanical vibrations. Under welding pressure, the vibration energy is transmitted to the welded interface through the sonotrode tip. Combined with ultrasonic shear stress, this energy induces high plastic deformation at the interface, removes impurities and oxide films, and creates a mechanical interlock. During ultrasonic welding, under the combined influence of pressure and ultrasound, the interface evolves with increasing temperature. It transitions from discontinuous local bond points to an entire initial bond surface. Metallurgical reactions, such as diffusion and dynamic recrystallization, further contribute to the formation of welded joints. The principle of ultrasonic welding is illustrated in Figure 1.

The main process parameters in ultrasonic welding include ultrasonic frequency, welding amplitude, welding pressure, ultrasonic power, and welding time. Among these, ultrasonic frequency is a fixed parameter, although a 5% loss typically occurs during electro-acoustic conversion, with approximately 1% attenuation after passing through the sonotrode tip. The welding amplitude is typically below 70 μm (peak-to-peak). The welding time affects the interface temperature, plastic deformation, and metallurgical bonding of the welding interface [19]. Welding pressure is a critical parameter that can be controlled carefully in ultrasonic welding, as it facilitates the transfer of mechanical vibration energy and induces plastic deformation at the interface. If the welding pressure is insufficient, the plastic deformation at the interface is inadequate to form a reliable bond. Conversely, excessive pressure may reduce the amplitude of the workpiece and cause cracks at the joint edges.

### 1.2. Application of Ultrasonic Welded Al/Cu Joint

**Figure 2:**
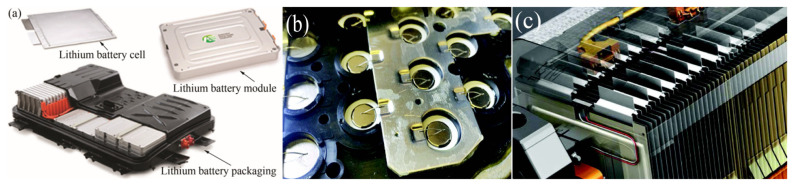
Application of Al/Cu joints: (**a**) Electric vehicle battery cells, modules and packs, (**b**) partial view of motor’s battery pack, (**c**) battery module.

The electric vehicle has great potential for decreasing air pollution and greenhouse gas emission [20]. As the driving source of new energy vehicles, the quality of power lithium battery packs determines the stability, power supply, and safety of electric vehicles [21]. Lithium battery packs consist of hundreds of single lithium batteries, with aluminum and copper used as the cathode and anode materials, respectively. Consequently, a large number of Al/Cu joints need to be welded during assembly, making Al/Cu welding critically important. Insufficient welding strength can lead to high internal resistance in the lithium battery pack, resulting in inadequate power supply for the vehicle and potential welded joint failure. During the welding process, the diffusion of aluminum and copper generates intermetallic compounds, whose resistance is significantly higher than that of the base metal. Therefore, the thickness of these intermetallic compounds must be strictly controlled. Excessively thin intermetallic compounds can pose serious safety risks, including a higher likelihood of lithium battery circuit short circuits or even explosions. Furthermore, joint deformation must also be tightly regulated. Excessive deformation can create assembly gaps in the lithium battery pack, potentially causing chemical leakage. Therefore, the welding quality of Al/Cu joints is critical to the performance and safety of lithium battery packs [22,23]. Compared to fusion welding methods, ultrasonic welding avoids metal melting, produces no spatter, and features energy-saving and environmentally friendly characteristics. Additionally, ultrasonic welding does not require surface treatment of the workpieces before welding [24]. These advantages have made ultrasonic welding the main welding method for bonding in lithium battery assembly. Figure 2 shows an application example of ultrasonic welding of Al/Cu in lithium batteries.

## 2. Research Progress of Experimentation of Aluminum and Copper Ultrasonic Welding

### 2.1. Welding Interface Temperature

**Figure 3:**
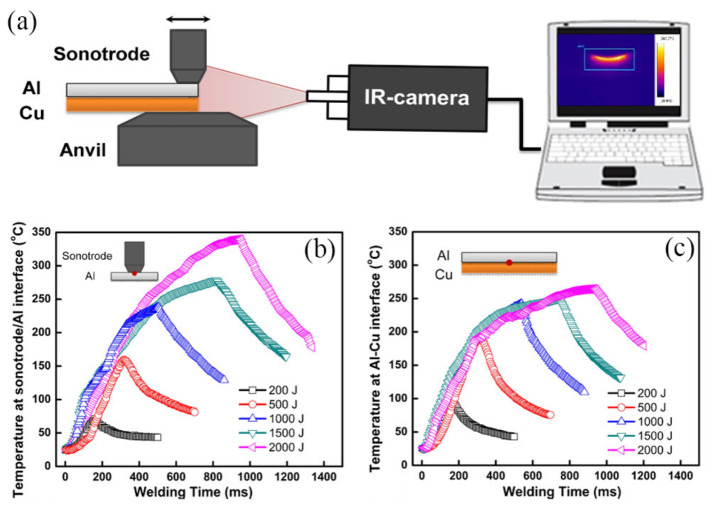
Infrared temperature measurement devices and temperature measurement results. (**a**) Infrared measurement device; (**b**) temperature at sonotrode/Al interface and (**c**) at Al/Cu interface [26].

**Figure 4:**
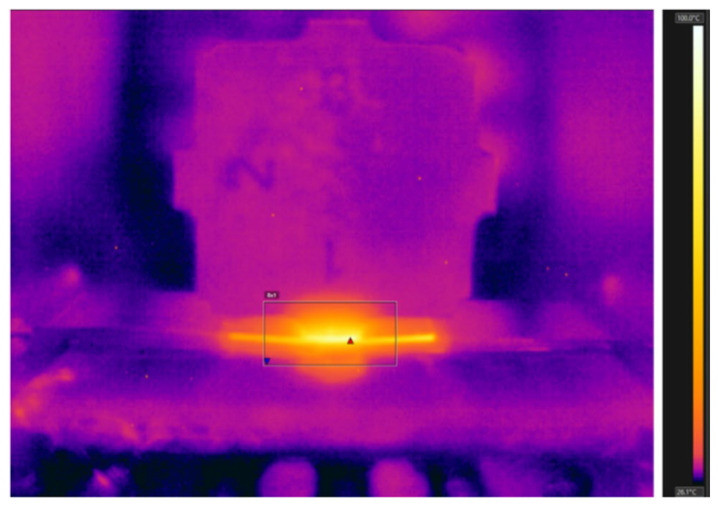
Infrared image captured at the weld zone with varied temperature distribution along the joint line [27].

The interface temperature in ultrasonic welding determines the metallurgical reactions at the interface, which significantly affect the quality of the joint [25]. At present, the interface temperature in ultrasonic welding is primarily measured using thermocouples or infrared thermometers. Zhao et al. [26] measured the interface temperature of a 6061-T6 aluminum alloy/pure copper interface using infrared thermography, the experimental set-up for the thermal measurements is shown in Figure 3a. It was observed that when the welding energy (multiply ultrasonic power by welding time) was less than 1000 J, the interface temperature was higher than that at the welding head/aluminum alloy interface. As the welding energy increased, the temperature at the sonotrode/upper aluminum alloy interface (Figure 3b) became higher than the Al/Cu interface temperature (Figure 3c). This was attributed to significant frictional heat generated at the interface between the sonotrode and aluminum alloy. De et al. [27] used an infrared thermal imager to measure the interface temperature of A1050-H16 aluminum/pure copper joints. Their results, shown in Figure 4, indicated that the primary heat generation occurred in the upper specimen beneath the sonotrode tip.

Fujii et al. [28] measured the interface temperature using a K-type thermocouple and reported a highest interface temperature of 223 °C. The interface temperature measured using a thermocouple embedded between the Al alloy and Cu sheets. Yang et al. [29] also used a K-type thermocouple to measure the interface temperature and found that the peak temperature reached 510 °C when the welding time was 0.7 s. This value was below the eutectic reaction temperature of the Cu specimen, but localized melting occurred near the interface. This phenomenon was attributed to the temperature measured by the thermocouples, which is just the average temperature of the weld region that cannot represent the actual temperature of the local area of the interface. However, the presence of a thermocouple at the welding interface is a foreign object with different physical properties that may produce an unknown effect. Specifically, the thermocouple can absorb a portion of the welding energy, potentially altering the thermal dynamics at the interface. While thinner thermocouple wires generally enhance temperature measurement accuracy due to their reduced thermal mass and faster response times, excessively thin wires are prone to fracture under significant plastic deformation, such as that caused by mechanical interlocking during welding. Furthermore, the necessity of drilling holes in the workpiece surface to accommodate thermocouple wires may introduce localized stress concentrations, thereby slightly compromising the mechanical performance of the joint.

In summary, for ultrasonic welding of aluminum and copper, the interface temperature for well-formed joints is typically 50–75% of the melting point of the aluminum alloy [17]. This temperature exceeds the critical threshold for dynamic recrystallization of the workpiece, ensuring sufficient plastic deformation at the interface to form a sound welding joint.

### 2.2. Interfacial Plastic Deformation

**Figure 5:**
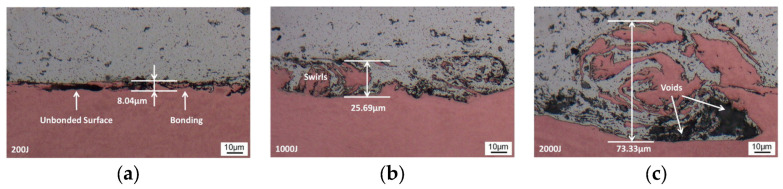
Microstructure of welding interface at different energies: (**a**) 200 J; (**b**) 500 J; and (**c**) 2000 J [26].

In the ultrasonic welding of Al/Cu, the plastic deformation at the interface breaks up the oxide film layer, causing the initial metal–metal contact to form and metallurgical reactions to occur. In addition, sufficient strain energy was stored to enable dynamic recrystallization due to deformation. Thus, the plastic deformation determines the formation and metallurgical reactions at the interface, making it a critical physical phenomenon in the welding process. Zhao et al. [26] investigated the plastic deformation process at the interface of 6061 aluminum alloy/pure copper during high-power ultrasonic welding under various welding energies. At lower welding energies, only a small number of micro-bonds were observed at the welding interface (Figure 5a). When the welding energy reached 1000 J, vortex-like plastic deformation appeared at the interface (Figure 5b), indicating mechanical interlocking and optimal joint quality. However, when the welding energy exceeded 2000 J, a significantly thick intermetallic compound formed at the interface (Figure 5c), which degraded joint quality.

**Figure 6:**
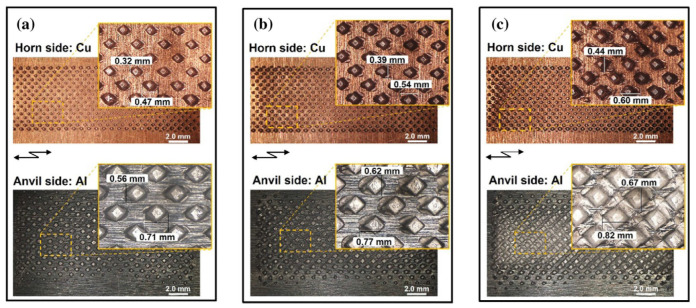
Surface images with horn/anvil tip imprints taken from the horn side (Cu sheet) and anvil side (Al sheet) of selected sets after ultrasonic welding. (**a**) 0.4 s, 50%; (**b**) 0.8 s, 70%; (**c**) 1.2 s, 90%. Vibration direction was applied in the horizontal direction of the images [20].

Shin et al. [20] studied the plastic deformation of specimens under different welding parameters. It was found that as welding time and vibration amplitude increased, the penetration depth of the sonotrode and anvil tip into the surfaces of the pure copper and 6061 aluminum alloy also increased. Since aluminum has lower hardness compared to copper, a greater embedding depth was observed on the aluminum alloy surface. At the end of the welding process, no visible cracks were detected at the joint, indicating satisfactory welding quality, as shown in Figure 6.

Fujii et al. [28] observed the migration of the oxide layer and brittle fracture phenomena at the interface of 1050 aluminum alloy/pure copper ultrasonic welding. During the welding process, the oxide layer was broken, and Cu particles were detected on the Al side. This phenomenon was attributed to friction and heat generation caused by plastic deformation, which induced material flow. When the welding time reached 0.2 s, the Al_2_O_3_ film migrated to the distal welding area. By extending the welding time to 0.4 s, aluminum oxide particles traveled 44 µm into the Al side and were eventually removed from the welding area during the final welding stage, promoting the formation of an initial metal-to-metal bond.

**Figure 7:**
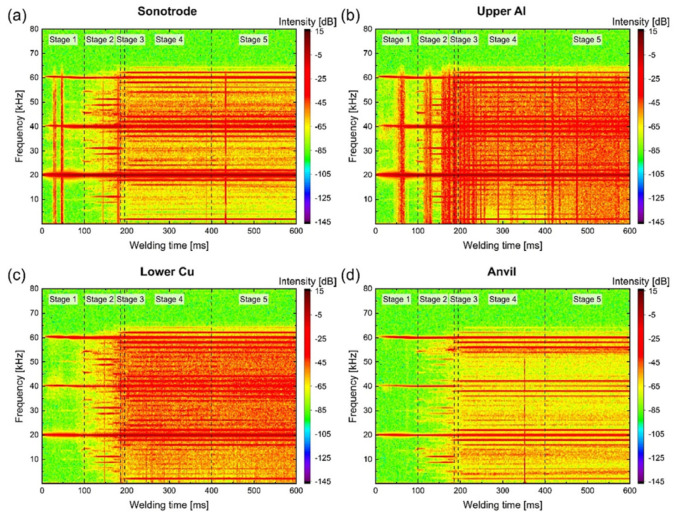
Time-frequency spectrograms of the velocity signal for (**a**) sonotrode; (**b**) upper Al; (**c**) lower Cu; (**d**) anvil [30].

Li et al. [30] measured the plastic deformation distribution of 1050 pure aluminum and CW008A-R240 copper alloy using a laser doppler vibrometer during ultrasonic welding. It was observed that plastic deformation occurred on the aluminum side during the early stage of the welding process (0–100 ms), resulting in distinct tooth tip marks (Figure 7a,b). At a welding time of 100 ms, both aluminum and copper experienced plastic deformation (Figure 7c,d), leading to the formation of a micro-weld.

**Figure 8:**
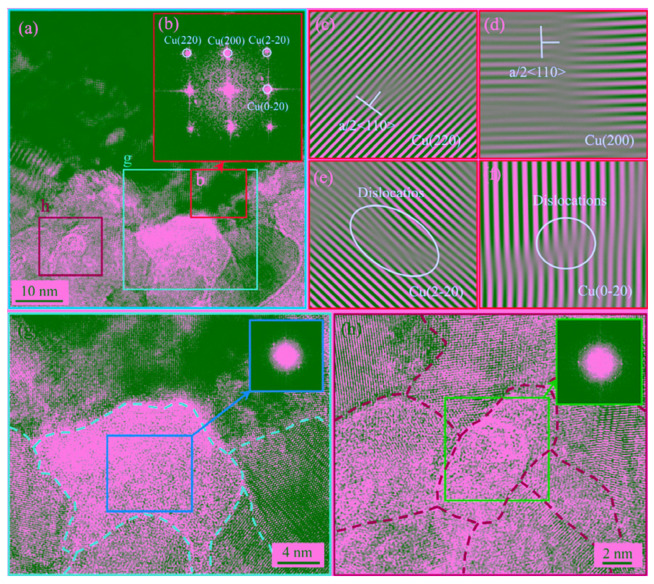
Morphology of microstructures at the interface. (**a**) amorphous regions; (**b**) inverse fast Fourier transform (IFFT) image of region (**b**); (**c**) IFFT image of (2 2 0); (**d**) IFFT image of (2 0 0); (**e**) IFFT image of (2–20); (**f**) IFFT image of (0–20); (**g**) orange boxed region in (**a**); (**h**) blue boxed region in (**a**) [31].

Cheng et al. [31] conducted ultrasonic welding of BVR2.5 Cu cables (2.5 mm² × 100 mm) and BLV6 Al cables (6.0 mm^2^ × 100 mm). Their study revealed the presence of a ~40 nm thick nanocrystal-amorphous mixed zone at the Al/Cu interface induced by ultrasonic welding. The formation of amorphous Al/Cu alloys was attributed to the increase in local free energy and shear stresses generated during ultrasonic vibration, as shown in Figure 8.

**Figure 9:**
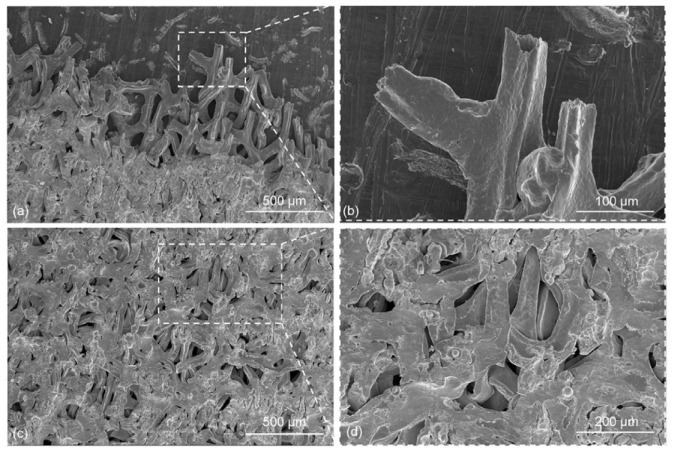
Micro-morphology of shear-tensile fracture of typical over-weld joint: (**a**) Cu foam fracture at the edge of the welding spot of the Al plate side × SED 200 times, (**b**) Cu foam fracture at the edge of the welding spot of the Al plate side × SED 1000 times, (**c**) Cu foam fracture at the welding spot of the Al plate side × SED 200 times, and (**d**) Cu foam fracture at the welding spot of the Al plate side × SED 500 times [32].

**Figure 10:**
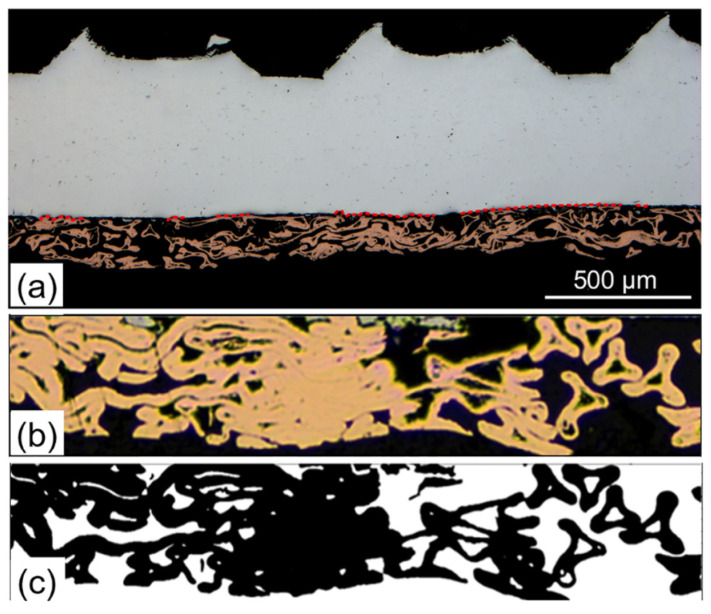
Schematic diagram of characteristic parameters of ultrasonic welding interface of Cu foam/Al plate: (**a**) measurement and extraction of interface morphology data, (**b**) interface morphology of Cu foam, and (**c**) extraction of interface morphology of skeleton structure of Cu foam [32].

Feng et al. [32] conducted ultrasonic welding of foam copper and pure aluminum. The morphological image of foam copper on the joint’s cross-section was extracted, enabling the estimation of foam copper porosity. The experimental results (Figure 9b) and prediction results (Figure 9c) show that the volume of foam copper occupied more than 60% of the space of the lower workpiece. In addition, the fracture morphology of the joint after over-welding was observed. It was found that the residual area of foam copper slightly exceeded the welding area (Figure 10a,b). Severe compression and deformation were detected on the foam copper side (Figure 10c), resulting in the near-complete filling of pores within the foam copper.

In summary, the plastic deformation mechanisms in aluminum and copper ultrasonic welding primarily encompass three distinct phenomena: tool head penetration into the workpiece, vortex-like interfacial plastic deformation, and mechanical interlocking formation. The penetration of the sonotrode into the upper specimen surface generates a series of interfacial bonding points, which significantly enhance the energy transfer efficiency of ultrasonic vibrations from the sonotrode to the welding interface. Regarding interfacial deformation, a strong correlation exists between the degree of vortex-like plastic deformation and joint strength, where moderate deformation promotes welding strength. However, it should be noted that excessive vortex-like deformation may compromise the metallurgical bonding quality, consequently deteriorating the joint performances. Among these mechanisms, mechanical interlocking plays a crucial role in improving the tensile-shear strength of the joint, representing a particularly significant and widely observed plastic deformation characteristic in ultrasonic welding processes.

### 2.3. Interfacial Grain Structures

**Figure 11:**
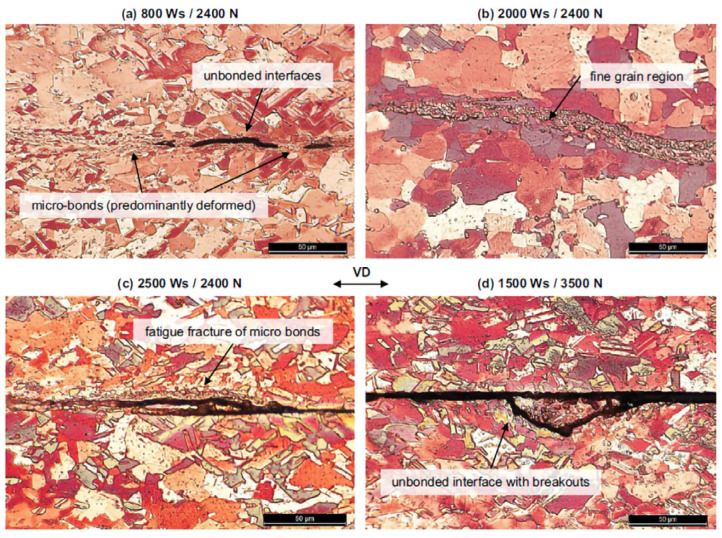
Micrographs of cross-sectioned CW-008A welded specimen with different weld energy and clamping force. (**a**) 800 Ws, 2400 N; (**b**) 2000 Ws, 2400 N; (**c**) 2500 Ws, 2400 N; (**d**) 1500 Ws, 3500 N [33].

**Figure 12:**
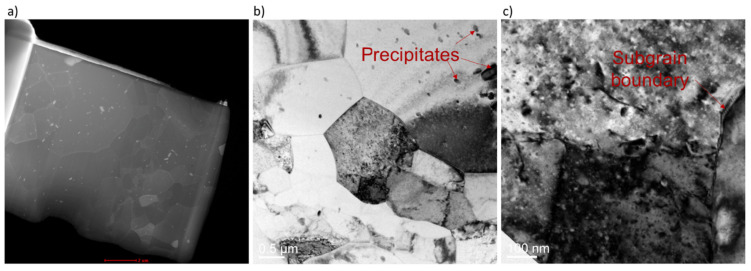
Electron microscopy of specimens. (**a**) Scanning transmission electron microscopy image of Al6061 wire deformed with 210 J/m^3^ ultrasonic energy density, (**b**) bright field TEM image of the same sample showing precipitates, and (**c**) bright field transmission electron microscope image showing sub-grain boundary [34].

Balz et al. [33] investigated the microstructures of the cross-section of AW-1050A aluminum/CW008A copper joints under different welding energies and clamping forces. It was found that low energy input resulted in bonding in micro-regions (Figure 11a), characterized by significant shear deformation of grains. In unbonded areas, only coarse grains from the base metal were observed. As welding energy increased, the length of the bonded area also increased (Figure 12b), enhancing the joint strength. However, excessive welding energy caused significant plastic deformation in the welding area (Figure 11c), reducing joint strength and leading to fatigue cracks caused by ultrasonic vibration. Additionally, under excessive clamping force, a large unbonded area was observed (Figure 11d), accompanied by local fractures or cracks on the surface of the specimen.

For Al/Cu ultrasonic welding, dynamic recrystallization at the interface does not alter the phase composition but significantly affects the interfacial microstructure distribution, which, in turn, affects the quality of ultrasonic welding. Deshpande et al. [34] observed recrystallization in 6061 aluminum alloy/pure copper ultrasonic welding. The precipitation of Mg_2_Si in the aluminum alloy was found to hinder dislocation movement, causing dislocation pile-up (Figure 12b) and the formation of new dislocations. Interactions between local dislocations and dislocation pile-up led to the formation of sub-grain boundaries (Figure 12c).

**Figure 13:**
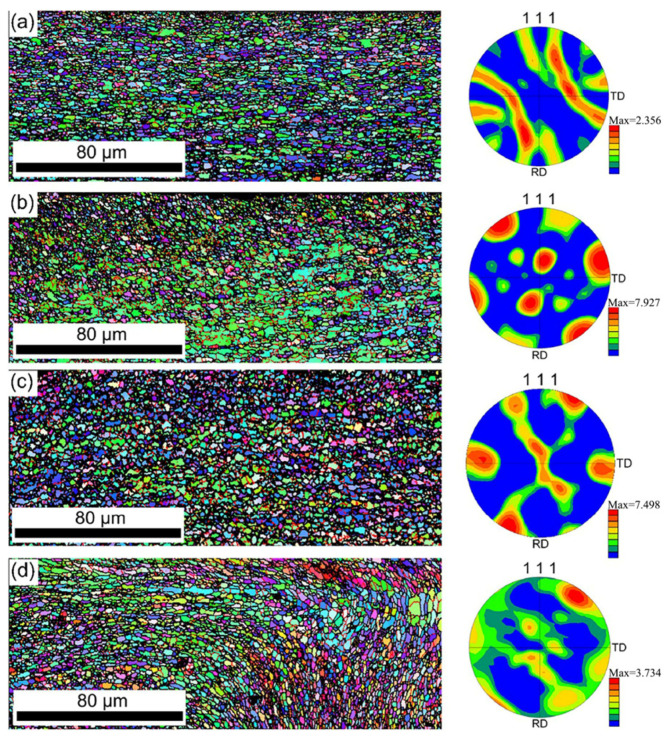
Resistance backscatter diffraction pattern and corresponding inverse pole pattern of Al side at different welding times of (**a**) 0.09 s; (**b**) 0.17 s; (**c**) 0.22 s and (**d**) 0.29 s [35].

Ma et al. [35] observed the microstructure evolution of the T2 copper/1060 aluminum alloy ultrasonic welding interface using electron backscatter diffraction. At a welding time of 0.09 s, no texture was observed. As the welding progressed, vortex-like deformation resulted in the appearance of a {110}<112> texture, indicating that plastic deformation dominated the initial stage of welding. At a welding time of 0.22 s, the interface structure consisted of dynamically recrystallized equiaxed grains with an average size of 3 μm. However, after a welding time of 0.29 s, ultrasonic shear deformation caused the texture to rotate, forming vortex-like grains without clear orientation (Figure 13).

**Figure 14:**
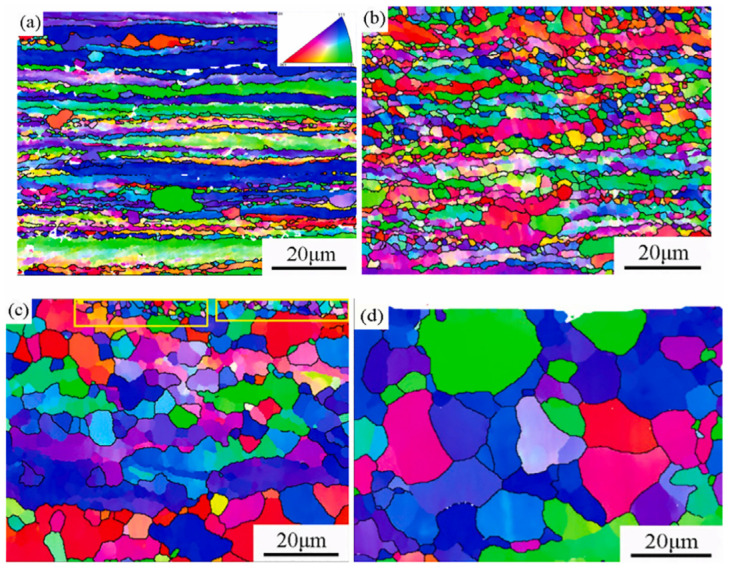
The grain orientation of aluminum alloy at the welding time of (**a**) 0 s; (**b**) 0.3 s; (**c**) 0.5 s and (**d**) 0.6 s [36].

Liu et al. [36] reported that after ultrasonic welding, the grain morphology and size on the pure copper side remained unchanged, while the 6061-T6 aluminum side showed significant microstructural changes (Figure 14). Under the combined action of welding pressure and ultrasonic vibration, slip dislocations occurred in the aluminum alloy, resulting in significant plastic deformation. Serrated grain boundaries interacted with wavy grain boundaries, causing long grains to pinch and form smaller grains. Dislocations absorbing ultrasonic energy led to the formation of a large number of small-angle grain boundaries, indicating dynamic recrystallization. After welding, the aluminum alloy primarily consisted of equiaxed grains.

**Figure 15:**
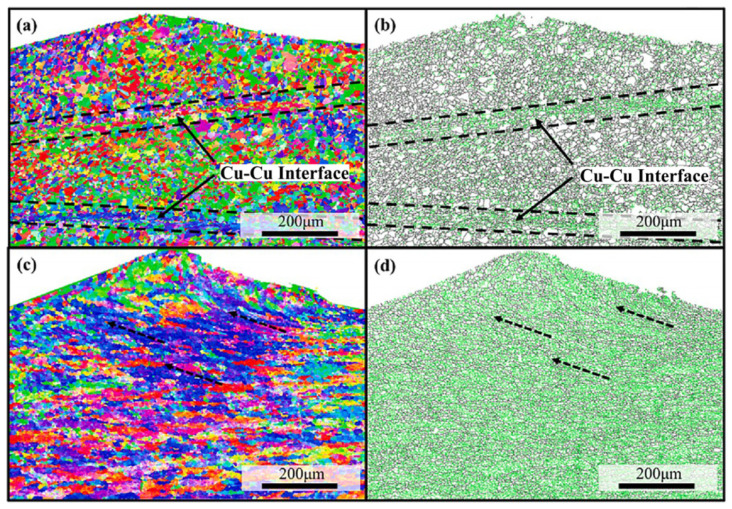
Recrystallized Cu and Al grains. (**a**) IPF at the peak of Cu; (**b**) grain boundary map at the peak of Cu; (**c**) IPF at the peak of Al; (**d**) grain boundary map at the peak of Al [31].

Cheng et al. [31] performed ultrasonic welding of BVR2.5 Cu and BLV6 Al cables to examine microstructural evolution and the mechanical properties of the joint. It was observed that the texture unit {001}<110> reformed as Cu grains rotated along the <110> crystal direction. Meanwhile, Al grains were fragmented into ultrafine equiaxed grains (~1.9 μm). Recrystallized Cu grains showed no strong texture, while Al grains grew with a strong Brass {110}<112> texture, as shown in Figure 15.

### 2.4. Interfacial Diffusion

**Figure 16:**
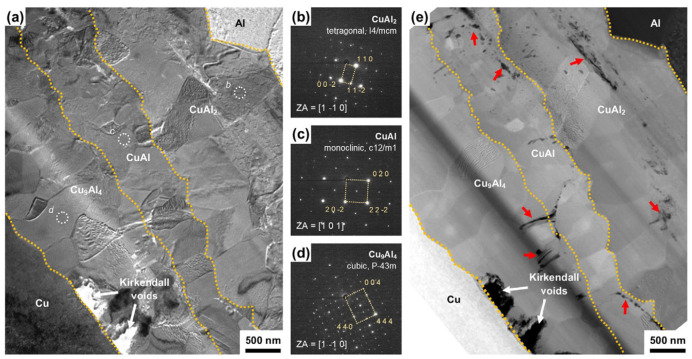
TEM images of Al/Cu interface. (**a**) TEM bright field (BF) micrograph of the Al/Cu interface after thermal aging at 200 °C for 700 h, (**b**–**d**) corresponding selected area electron diffraction (SAED) patterns for b CuAl_2_, (**c**) CuAl and d Cu_9_Al_4_ intermetallic compound layers, and (**e**) scanning TEM (STEM) high-angle annular dark field image of the Al/Cu (same area with TEM BF image in (**a**)) [37].

The strength of Al/Cu ultrasonic welding joints largely depends on the intermetallic compounds formed through atomic diffusion. When the brittle intermediate compound is too thick, it deteriorates the quality of the joint, but when the brittle intermediate phase is too thin, the interface diffusion is insufficient to form a reliable joint. Jeong et al. [37] observed the interface diffusion behavior of 1050 Al and pure copper during ultrasonic welding. They observed that intermetallic compound layers, including CuAl_2_, CuAl, and Cu_9_Al_4_, were generated along the welding interface, as shown in Figure 16a–d. Micro-voids, marked by red arrows, were identified near the boundaries of Cu_9_Al_4_/Cu and Cu_9_Al_4_/CuAl (Figure 16e), which negatively impacted the joint’s performance.

**Figure 17:**
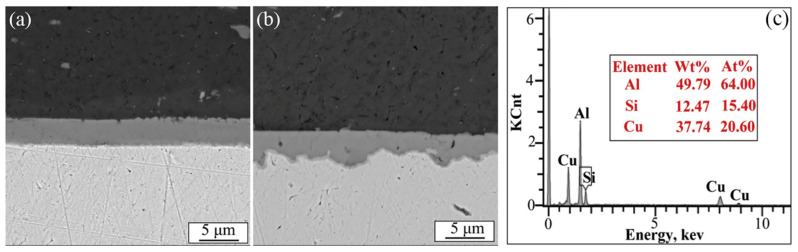
SEM results under different clamping force: (**a**) 1975 N, (**b**) 2175 N, (**c**) EDS chemical analysis under pressure of 1975 N [38].

Li et al. [38] investigated the effects of welding pressure on dynamic process and joint characteristics (strength and fracture) of high-power ultrasonic welding. It was found that the thickness of intermetallic compounds at the 6061-T6 aluminum/pure copper ultrasonic welding interface peaked at a clamping force of 1975 N (Figure 17a). At a clamping force of 2175 N, even though the temperature rose, the thickness of the intermetallic compound layer decreased (Figure 17b). This reduction was attributed to the impurity element Si, which, along with Al and Cu, forms a face-centered cubic crystal. At elevated temperatures, the softened material allows Si to diffuse into the Al/Cu interface, thereby limiting interface diffusion.

In summary, in ultrasonic welding of the Cu/Al joint, the bonding quality is primarily governed by two critical factors: vacancy concentration and interfacial temperature, both of which are directly influenced by the ultrasonic power. The experimental evidence suggests that increased ultrasonic power leads to enhanced atomic diffusion, resulting in a thicker diffusion layer at the weld interface. Particularly in the case of Cu/Al joints, the presence of identical face-centered cubic (FCC) crystal structures in both materials facilitate the generation of extensive point defects within the crystal lattice. This structural compatibility significantly enhances atomic diffusion rates, thereby promoting the formation of a robust metallurgical bond at the interface.

### 2.5. Effects of the Metal Interlayer on Ultrasonic Welding of Aluminum to Copper

**Figure 18:**
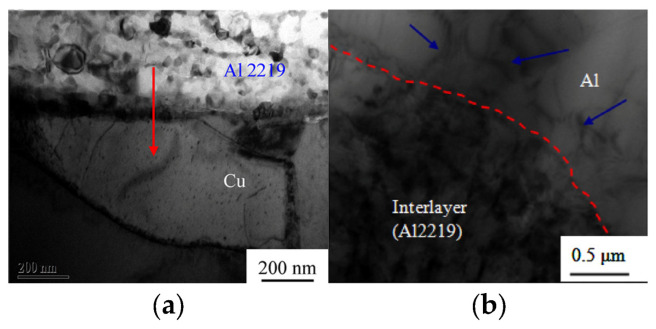
TEM and EDS analysis of the Al/Cu joint with interlayer at welding time of 0.5 s: (**a**) the Al/interlayer interface [39], (**b**) the Cu/interlayer interface [40].

Ni et al. [39] added 2219 aluminum alloy powder to the ultrasonic welding interface of pure copper/1100 aluminum alloy to enhance metallurgical behavior. At a welding energy of 1500 J, no intermetallic compounds were formed at the welding interface (Figure 18a). It was suggested that the addition of Al2219 alloy caused plastic deformation of the metal, resulting in more effective contact, which suppressed the formation of the intermetallic compound layer. In addition, the incorporation of 2219 aluminum alloy powder produced an uneven welding interface [40], as shown in Figure 18b.

**Figure 19:**
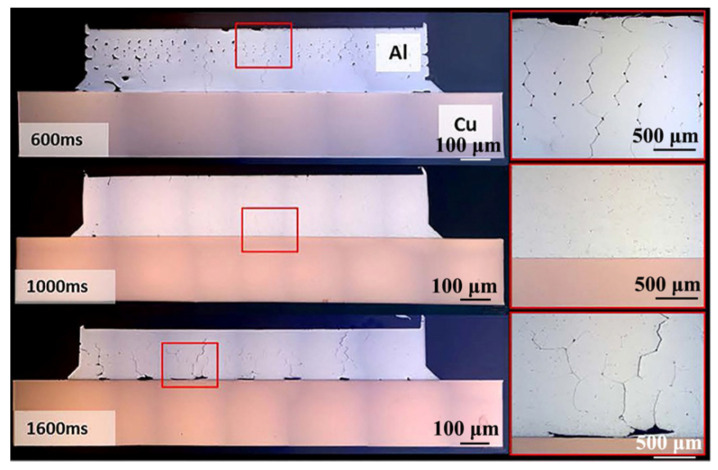
Weld cross-sections of samples with Ni-plated terminals at increasing welding time [41].

Bergmann et al. [41] investigated the effect of pure Ni coating on ultrasonic welding of Al/Cu. As shown in Figure 19, a significant number of unwelded areas were observed on the aluminum side at a welding time of 0.6 s. As the welding time increased to 1 s, the joint was well-formed. However, at 1.8 s without the pure Ni coating layer, the joint exhibited lower strength due to the presence of visible pores.

**Figure 20:**
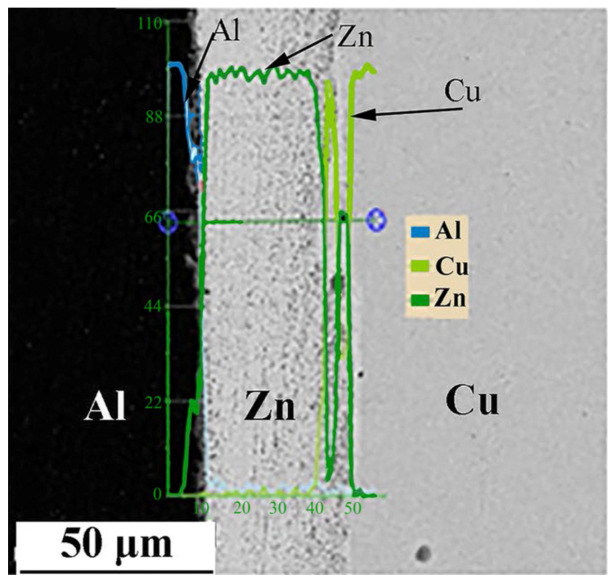
Intermetallic compound profile of interface in USW with Zn interlayer [42].

Li et al. [42] investigated the effect of a Zn interlayer on pure Al and copper ultrasonic welding. It was observed that the strength of the welded joint with the zinc layer was 115% higher than that without the zinc layer at a welding energy of 700 J. This improvement was attributed to the formation of an intermetallic compound layer of Cu_5_Zn_8_ (Figure 20), which enhanced the joint strength.

In summary, the use of a metal interlayer in Cu/Al ultrasonic welding can effectively reduce the formation of brittle intermetallic compounds, improve welding quality, and enhance process stability. However, it also introduces additional costs, potential metallurgical reaction issues at the interlayer/specimen interfaces, and requires precise control over the thickness of the interlayer.

### 2.6. Mechanical Properties of Ultrasonic Welded Al/Cu Joint

**Figure 21:**
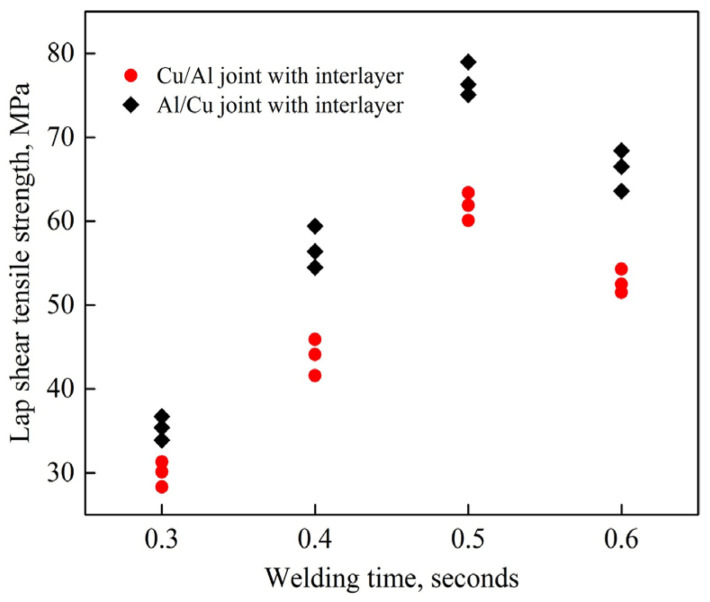
Lap shear tensile strength of the ultrasonically welded joints with different lap configurations [40].

Ni et al. [40] conducted ultrasonic welding of 1100 aluminum alloy/pure copper in different overlapping sequences. They found that the tensile strength of the joint is 27.3% higher when aluminum serves as the upper workpiece compared to when copper is positioned above (Figure 21). This enhancement is attributed to the higher interface temperature and plastic deformation with aluminum on top, which promotes better welding interface formation. However, when aluminum is used as the upper workpiece, the microhardness of the joint is reduced due to the higher interface temperature, which induces more recrystallization and grain growth, thereby softening the material, according to the study by Ji et al. [43].

Ao et al. [37] analyzed the effect of heat treatment temperature on the mechanical properties of ultrasonic welded joints of pure aluminum and pure copper. They discovered that exceeding a welding heat treatment temperature of 150 °C causes a rapid decrease in the maximum tensile load of the joint, owing to the formation and growth of the intermetallic compound layer, with a consequent shift in the fracture mode from pull-out to interfacial fracture. Satpathy et al. [14,44] predicted the strength of ultrasonic welded joints of pure aluminum and pure copper and found that welding pressure is the most critical factor influencing the peel strength of the joint, followed by welding time and vibration amplitude.

In summary, as ultrasonic power increases, welding strength improves significantly, but pressure and welding amplitude are interdependent, limiting joint strength optimization using methods like Taguchi or response surface. Future research should focus on optimizing welded joint quality based on material plastic deformation, which governs interfacial metallurgical reactions. AI technologies show great potential in enhancing Cu/Al ultrasonic welding quality. Real-time monitoring with high-resolution cameras captures precise welding deformation data, enabling detailed interface characterization through point position datasets and 3D surface contours. Advanced big data analytics and machine learning algorithms improve the accuracy of welding quality evaluation and prediction. Additionally, machine learning optimizes mesh element distribution in finite element modeling, enhancing simulation fidelity and predictive capabilities.

### 2.7. Ultrasonic Welding of Laminated Al/Cu Joints

The pouch lithium battery pack involves the welding of multiple layers of copper and aluminum joints. Unlike double-layer plate welding, multi-layer copper and aluminum joints demonstrate an uneven distribution of welding energy, leading to more complex metallurgical reactions at the interface.

**Figure 22:**
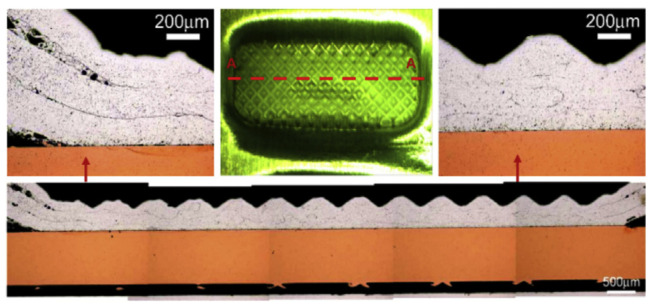
Cross-sectional optical microscope (OM) view of a normal-weld specimen sectioned along the long axis for the middle weld spot [45].

**Figure 23:**
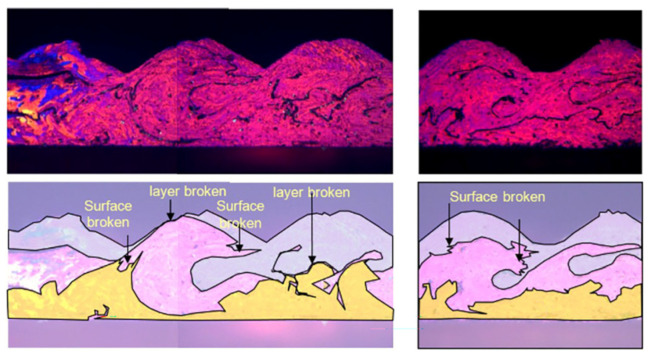
Sectioned and etched Al tabs interfaces observed by OM [45].

Wu et al. [45] conducted ultrasonic welding on three layers of pure aluminum plates and one layer of nickel-plated copper sheet at different welding energies. They observed that all three layers of aluminum plates deformed severely into a wavy shape (Figure 22), with the degree of deformation progressively decreasing from the top to the bottom layer. The aluminum layers exhibited significant metal flow and mechanical interlocking. While severe plastic deformation and material flow disrupted the original grain structure, the absence of significant new grains (Figure 23) indicated no recrystallization occurred. The copper side remained largely undeformed. This led to the conclusion that the ultrasonic welding mechanism for multi-layer boards is primarily a physical bonding effect caused by friction.

**Figure 24:**
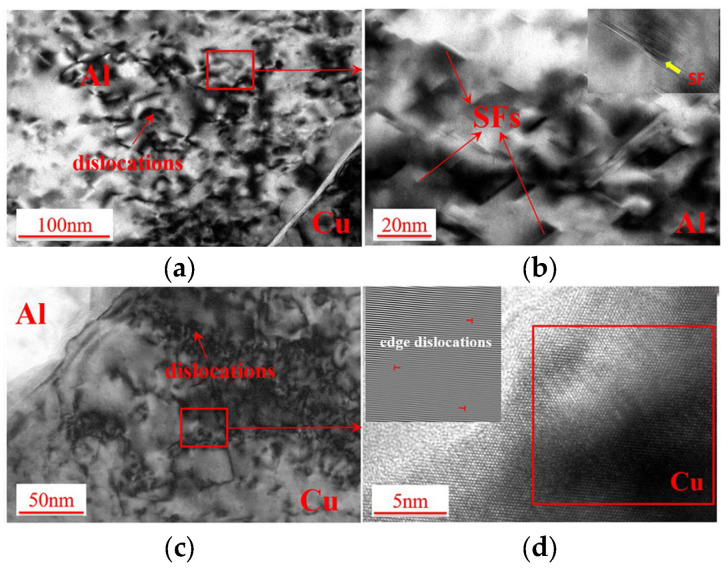
The structure of the Al and Cu grains near the interface observed under TEM dual beam conditions: (**a**) dislocations in Al grains near interface after welding; (**b**) stacking faults in Al grains near interface; (**c**) dislocations in Cu grains near interface after welding; (**d**) edge dislocations in Cu grains near interface [46].

Zhang et al. [46] observed the microstructural evolution of ultrasonic welding between 1100 aluminum and multi-layer pure copper plates before and after welding using TEM (Figure 24). Numerous dislocations were observed in the grains of the base metal near the welding area. High-density stacking faults were detected in the Al grains due to severe plastic deformation, and dislocations were activated towards the {111} 1/2<110> in the Cu grains, confirming substantial plastic deformation during ultrasonic welding. However, no significant dynamically recrystallized grains were present near the welding interface, contrasting with observations in the ultrasonic welding of Al and Cu sheets.

**Figure 25:**
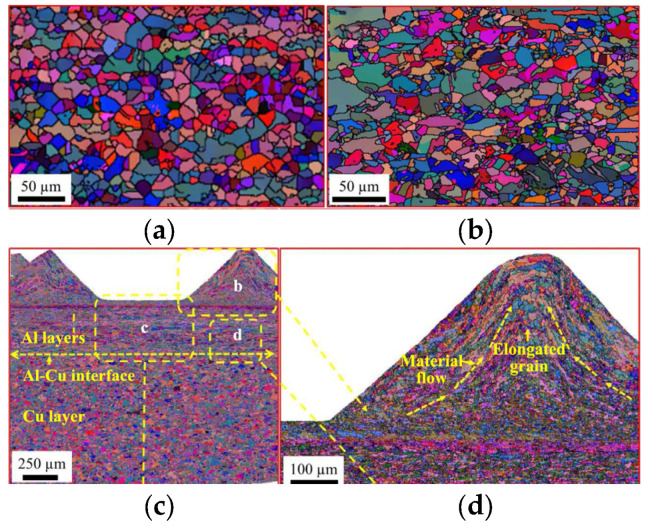
EBSD images of grains in different weld areas: (**a**) aluminum sheet before welding; (**b**) copper sheet before welding; (**c**) a slice at the center of weld zone; (**d**) the crest [47].

Dhara et al. [47] performed ultrasonic welding of 1050 aluminum alloy and multi-layer pure copper, reporting elongated grains after welding. Three distinct zones were identified in the aluminum layer: a severely deformed region with grains approximately 5 µm in size near the welding interface, a compressed welding zone due to deformation caused by the upper aluminum plate, and a middle zone containing elongated grains, as shown in Figure 25.

Shin et al. [20] evaluated the mechanical properties of ultrasonic welded joints between 1050 aluminum alloy and multi-layer C1220 copper sheets. It was determined that all joint fractures occurred at the interface. Aluminum’s higher tendency for severe plastic deformation resulted in a greater number of unconnected areas in the joint when aluminum served as the upper workpiece.

Das et al. [48] investigated the effects of welding pressure, amplitude, and welding time on the ultrasonic welding quality of 1050 aluminum alloy and multi-layer pure copper plates. Using response surface analysis optimization, the parameters that achieved maximum tensile shear strength were identified.

In summary, the joint strength of ultrasonically welded laminated Cu/Al joints exhibits a progressive decrease along the direction from the sonotrode to the anvil. The conventional ultrasonic welding process for manufacturing high-quality laminated aluminum–copper joints encounters substantial technical limitations, which necessitate either the utilization of higher-power ultrasonic transducers (minimum 7.0 kW) or the incorporation of additional heat sources to optimize the welding quality of these laminated structures.

### 2.8. Aluminum and Copper Resistance Assisted Ultrasonic Hybrid Welding

In single ultrasonic welding, the temperature increases and intermetallic compound growth during the welding process are solely influenced by ultrasonic vibration energy, making it challenging to improve the mechanical properties of the joint. Although studies have explored laser-assisted ultrasonic welding [49] and induction heat-assisted ultrasonic welding [50], the primary role of these heat sources is limited to softening the workpiece prior to welding. To address this limitation, Cao et al. proposed a resistance heat-assisted ultrasonic welding method, in which resistance heating and ultrasonic vibration are applied to the welding interface simultaneously. The mechanism involves resistance heating promoting material softening, thereby amplifying the effects of ultrasonic vibration. In addition, ultrasonic vibration breaks the oxide film and expands the current flow channel.

**Figure 26:**
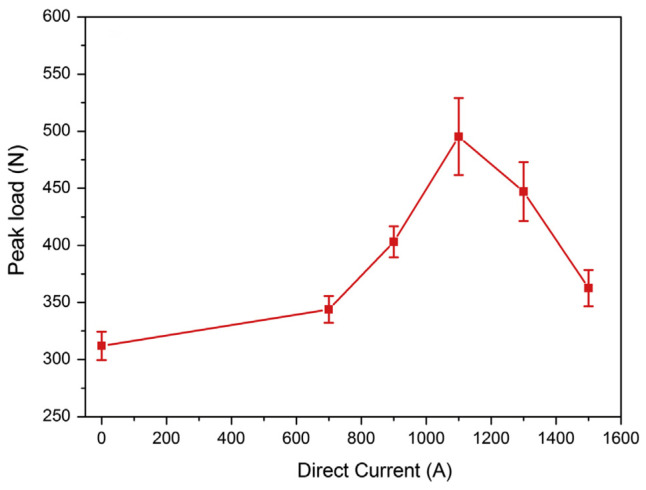
Tensile shear test results of low power ultrasonic welding joints under different currents [51].

**Figure 27:**
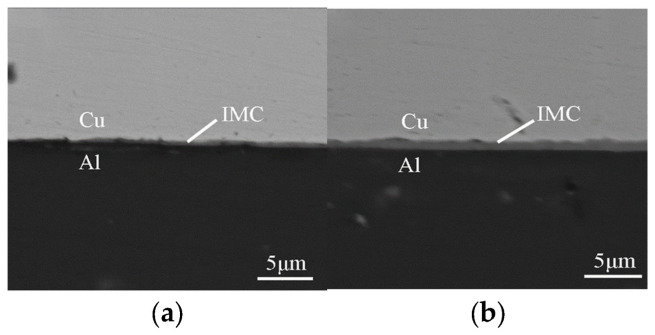
SEM images for intermetallic compound at the center of welding region with assisted current of (**a**) 2700 A; (**b**) 0 A [52].

Yang et al. [51] conducted resistance thermal-assisted ultrasonic welding on low-power pure copper/6061-T6 aluminum alloy. Their results demonstrated that resistance heating effectively facilitated interface metallurgical reactions (Figure 26), leading to a significant improvement in joint strength. Liu et al. [52] investigated resistance heat-assisted high-power ultrasonic welding of pure copper/6061-T6 aluminum alloy. They found that compared to single ultrasonic welding, the intermetallic compound layer produced by composite welding was thinner and exhibited a 10% higher quality under a 2700 A current with the same welding time (Figure 27).

In summary, the application of resistance heat-assisted ultrasonic welding technology has proven to significantly enhance the metallurgical bonding of the Al/Cu joints. This technological advancement shows promising potential for the successful fabrication of reliable multi-layer Al/Cu ultrasonic welding joints in future industrial applications.

## 3. Research Progress in Modeling of Ultrasonic Welding of Al/Cu

### 3.1. Finite Element Modeling of Ultrasonic Welding of Al/Cu

Ultrasonic welding is a dynamic and complex process, and experimental conditions often fail to fully explain the thermo-mechanical coupling process in welding. The finite element method can simulate these dynamic processes through appropriate simplifications. The following assumptions are made in elucidating the thermo-mechanical coupling process: The sonotrode that was used in this analysis had a uniform cross-sectional area at the tip, the area of weld is equal to the area of deformation zone owing to a large ultrasonic shear force at the interface; and there was no air gap between the two aluminum sheets. The room temperature is assumed to be uniform.

**Figure 28:**
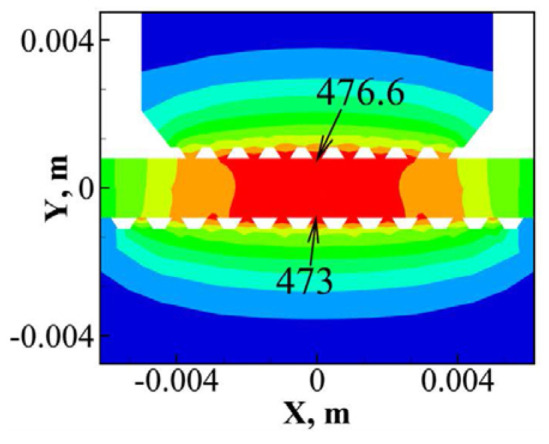
Temperature distribution at weld time 0.5 s [53].

**Figure 29:**
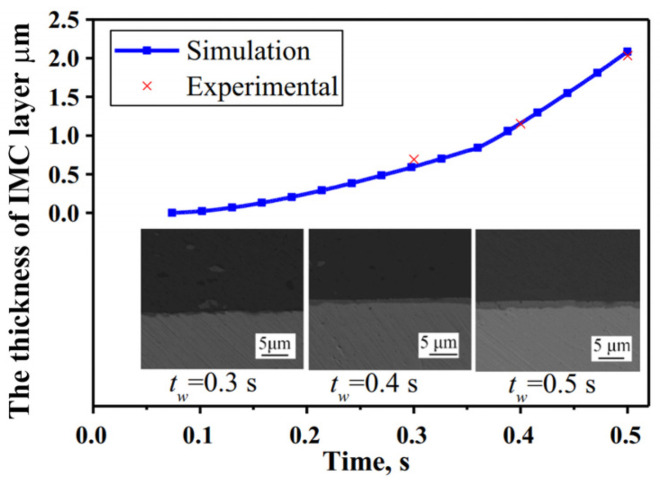
Comparison of simulated intermetallic compound thickness with experimental results [53].

Li et al. [53] established a three-dimensional finite element model of Al/Cu ultrasonic welding that incorporates the morphology of the sonotrode and anvil, as well as real-time dynamic material softening. Heat generation at the upper and bottom interfaces, dynamic material softening, and high convection boundary conditions were considered in this model. The model predicted a highest temperature of 476.6 °C at the contact surface between the welding head and the aluminum plate, while the highest temperature at the center of the welding interface was 449 °C (Figure 28). Using the simulated temperature results in conjunction with Fick’s diffusion law, the intermetallic compound thickness was calculated, demonstrating a nonlinear growth relationship, as shown in Figure 29.

**Figure 30:**
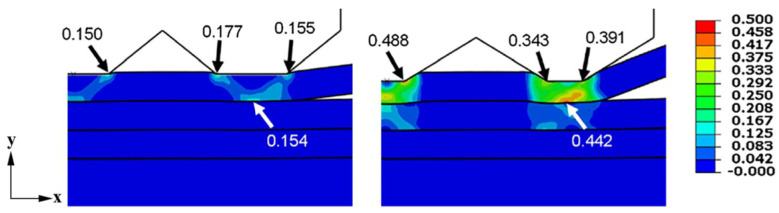
The maximum principal plastic strain [56].

Chen et al. [54] developed an Al/Cu ultrasonic welding model that accounts for ultrasonic softening. The simulation results indicated that only 20% of the total welding heat was generated by plastic deformation, with frictional heat being the main heat source. They also found that the downward displacement of the sonotrode tip increased significantly after acoustic softening effect (ASE) was considered [55]. Lee et al. [56] established a two-dimensional Al/Cu ultrasonic welding model and found that the penetration depth of the upper specimen exceeded that of the lower workpiece (Figure 30). The study also highlighted variations in the dynamic friction process caused by different sonotrode tip geometries. Horn designed with the size of the contact teeth of base horn can result in higher frictional energy dissipation rates than the base horn, and plastic energy dissipation rates are 5% or less than frictional energy dissipation rates.

**Figure 31:**
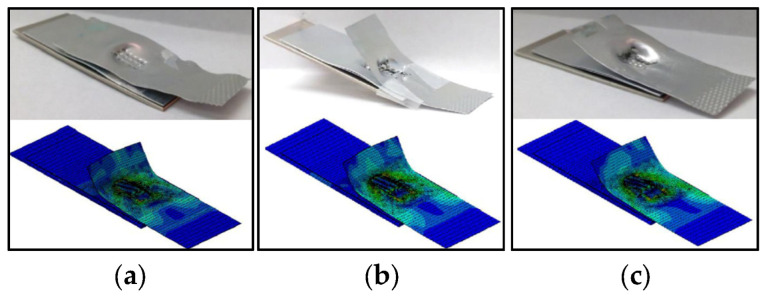
Comparison of the experimental and numerical results for four-layered welds: (**a**) underweld; (**b**) normal weld; (**c**) overweld [57].

Xi et al. [57] developed a finite element model for ultrasonic welding of Al/Al/Al/Cu four-layer laminates. The failure characteristics of multi-layer copper and aluminum joints were simulated by applying boundary conditions for temperature and stress fields, as well as considering the welding area and workpiece thickness as initial geometric conditions. As shown in Figure 31, interface failure, indicative of under-welding, was the primary failure mode at shorter welding times. Pull-out fractures, a sign of normal welding, are characterized by well-formed joints. Over-welding led to fractures within the welding area.

**Figure 32:**
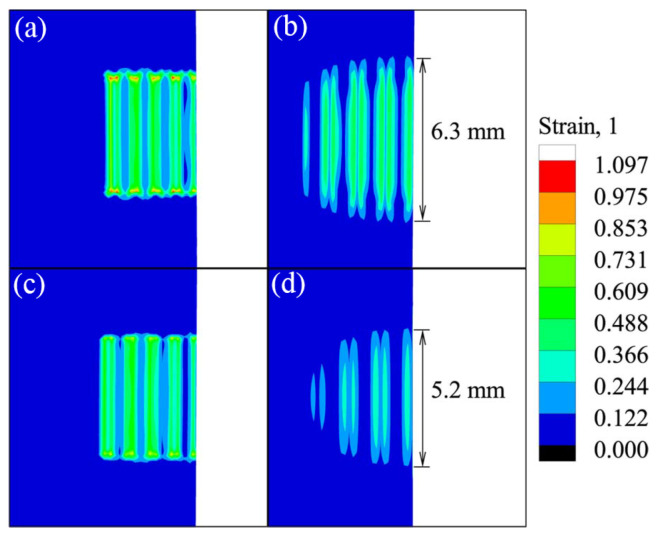
Plastic deformation distributions at welding time of 0.2 s on (**a**) top surface for RUSW; (**b**) bottom surface for RUSW; (**c**) top surface for USW; (**d**) bottom surface for ultrasonic welding [58].

Li et al. [58] established a finite element model for Al/Cu resistance heat-assisted high-power ultrasonic welding (RUSW) to investigate the temperature field and plastic strain distribution during the hybrid welding process. Dynamic ultrasonic softening and heat generation related to the vibration amplitudes of specimens and sonotrode were considered in the model. The findings revealed that within the same duration of 0.2 s, resistance heating significantly improved the welding quality and enhanced material plastic deformation (Figure 32).

In summary, the finite element method demonstrates its capability in predicting the macroscopic thermo-mechanical coupling field during ultrasonic welding of copper and aluminum. However, the computational accuracy of current models faces significant challenges due to the limited understanding of the friction process at the welding interface. This limitation has led to the necessity of employing assumptions or substantial simplifications regarding the friction coefficient in existing finite element models.

### 3.2. Molecular Dynamics Simulation

Although the finite element simulation method can explain the macroscopic thermal-structural coupling mechanism in ultrasonic welding, it is unable to explore other welding mechanisms at the atomic level. The molecular dynamics (MD) method offers significant advantages in uncovering atomic-scale defect evolution and explaining microscopic diffusion phenomena. Since the initial formation and fracture of ultrasonic welding joints occur at the Å level, MD simulations are particularly effective in explaining metallurgical phenomena in welding.

**Figure 33:**
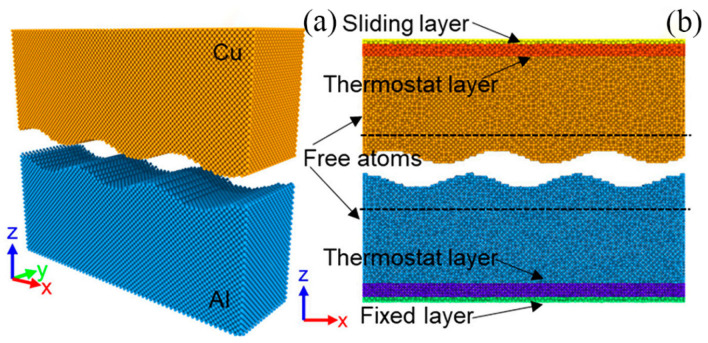
MD simulation model used for Al and Cu ultrasonic welding: (**a**) Initial configuration of sample; (**b**) Equilibrated configuration after relaxation under zero pressure at 300 K [59].

Yang et al. [59] established a three-dimensional MD simulation model for Al/Cu ultrasonic welding (Figure 33). The simulation results revealed that during the ultrasonic welding process, diffusion at the welding interface was asymmetrically distributed, with aluminum migrating at a much faster rate than copper. Under the influence of ultrasonic stress, the microstructure of the welding interface transitions from a regular face-centered cubic structure to a disordered arrangement, with the degree of disorder increasing as welding time progresses.

**Figure 34:**
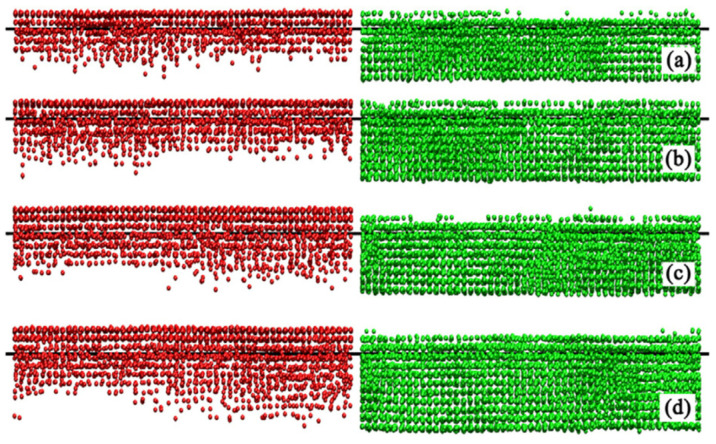
Interdiffusion of copper (red) and aluminum atoms (green) at different temperatures of (**a**) 650 K; (**b**) 700 K; (**c**) 750 K; and (**d**) 800 K [60].

Li et al. [60] used MD simulations combined with the embedded atomic method (EAM) to study the effect of temperature on interface diffusion in ultrasonic welding of pure aluminum and single-crystal copper. The results indicated that the thickness of the diffusion layer increases as temperature rises. Moreover, the diffusion mainly involves copper atoms diffusing into aluminum, with minimal aluminum atom diffusion into the interior of copper, as shown in Figure 34. This behavior is attributed to the significantly higher melting point and stronger bond energy of copper compared to aluminum, making copper bonds more difficult to break and form vacancies and thus resulting in aluminum migrating at a much faster rate than copper. In contrast, aluminum bonds are more prone to breakage, enabling aluminum atoms to move easily and create vacancies.

**Figure 35:**
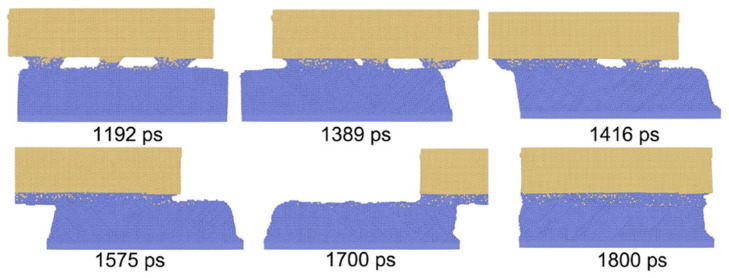
Asperity flattening and weld formation [35].

Ma et al. [35] developed a three-dimensional MD model to predict the evolution of material dislocations during the welding process. The model defined each workpiece material as consisting of a 10 Å sliding layer, a thermal layer, and a Newtonian layer. The sliding layer is associated with driving forces and displacement, while the thermal layer is related only to temperature. It was observed that micro-bonds initially form through protruding atomic contacts (Figure 35). Consequently, it was concluded that reducing surface roughness during ultrasonic welding can accelerate the formation of welded joints.

**Figure 36:**
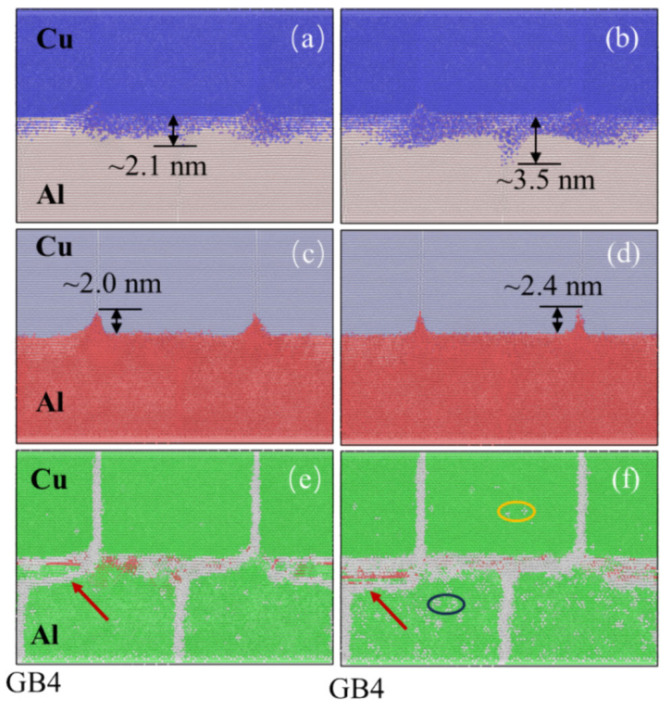
Snapshot of Cu atoms diffused into Al matrix (partially transparent). (**a**,**b**) Al atoms diffused into Cu matrix (partially transparent) (**c**,**d**) and interfacial structures (**e**,**f**) at end of welding [61].

Yang et al. [61] investigated diffusion processes in bi-crystalline Al/Cu joints using molecular dynamics simulations. Their findings demonstrated that GBs facilitated the diffusion of both Cu and Al atoms, with Cu atoms predominantly migrating into the Al matrix. The shearing effect during the ultrasonic welding process, combined with GB sliding, was found to induce grain recrystallization at the interface, as shown in Figure 36.

**Figure 37:**
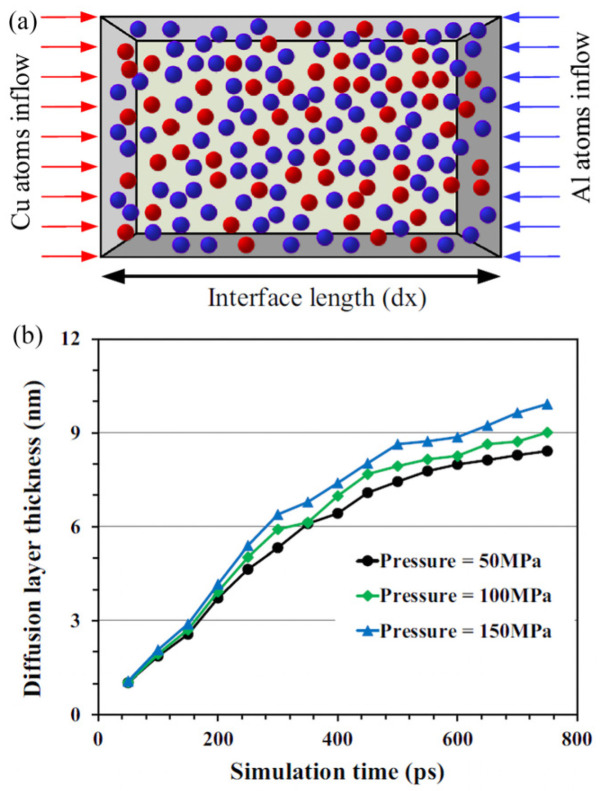
Molecular mechanics model (**a**) and diffusion layer thickness at different pressures (**b**) [62].

Samanta et al. [62] investigated the diffusion behavior of aluminum and multi-layer copper plates during ultrasonic welding using the MD method (Figure 37a). They revealed that the diffusion rate increases with higher pressure (Figure 37b), and greater pressure resulted in thicker diffusion layers, indicating a strong relationship between pressure and diffusion rate.

In summary, while molecular dynamics (MD) simulations provide a powerful approach for investigating the microscopic mechanisms of ultrasonic welding, they face several inherent limitations. First, the timescales of ultrasonic welding processes far exceed the femtosecond-level time steps used in MD simulations, making it challenging to capture the complete welding cycle. Second, MD simulations are constrained to a finite number of atoms, typically at the nanoscale, which prevents direct simulation of macroscopic welding processes. Third, the accuracy of simulation results relies heavily on the precision of the mechanical fields, and the existing field models may not fully account for the complex atomic interactions during ultrasonic welding.

Additionally, ultrasonic welding involves intricate boundary conditions, such as high-frequency vibrations and non-uniform stress distributions, which are difficult to accurately represent in MD simulations. Moreover, validating MD simulation results experimentally remains a significant challenge, particularly at the nanoscale, where obtaining precise experimental data is inherently difficult. Finally, the complex interfacial behavior in ultrasonic welding, including atomic-level interactions and energy transfer mechanisms at interfaces, poses substantial challenges for accurate representation in MD simulations.

## 4. Conclusions

(1) In Al/Cu ultrasonic welding, the intermetallic compounds generated by interface diffusion are primarily brittle AlCu and Al_2_Cu. The addition of auxiliary metals such as Zn and Al alters the chemical composition of the intermetallic compounds, suppressing the formation of brittle phases and promoting metallurgical bonding at the interface. The intermetallic compound layer significantly influences the welding quality of Al/Cu joints. Predicting and controlling the growth of the intermetallic compound layer remains a critical research focus.
(2) The deformation mechanism in ultrasonic welding is not yet fully understood. The vortex-like appearance at the interface is believed to result from the uneven distribution of plastic deformation. Since plastic deformation primarily arises from the ultrasonic softening of the workpiece, numerical simulations that integrate the sound field intensity distribution with the temperature field distribution can be used to predict this vortex-like plastic deformation. This approach offers a better understanding of the interface plastic deformation mechanism.
(3) As ultrasonic power increases, the welding strength improves significantly. However, the pressure and welding amplitude are interdependent parameters. Consequently, the improvement in joint strength corresponding to welding parameters optimized using the Taguchi method or the response surface method is limited. Since the plastic deformation of the workpiece governs the metallurgical reaction at the interface, it is a feasible research direction in the future to optimize the quality of welded joints according to the plastic deformation of materials.
(4) Numerical simulations of the temperature field and macroscopic plastic deformation using the finite element method, as well as simulations of microscopic defects and deformation using the molecular dynamics method, are the primary areas of research in this domain. However, the predicted plastic strain of the interface based on current models is significantly lower than the actual welding strain. Developing a nanoscale finite element model that accounts for ultrasonic softening could more accurately predict the microscopic plastic strain of materials. Moreover, joint formation arises from the dynamic plastic flow of materials. Due to the limited understanding of the influence of ultrasound on the viscosity coefficients of aluminum and copper, no reports are available on the simulation of plastic flow behavior. A fluid model that incorporates the effects of ultrasound on viscosity coefficients could effectively predict joint formation.
(5) The integration of artificial intelligence (AI) technologies has demonstrated significant potential in enhancing the quality of Cu/Al ultrasonic welding processes. Through the implementation of real-time monitoring systems utilizing high-resolution cameras, precise measurements of welding deformation can be captured and analyzed. This data acquisition process enables the generation of comprehensive point position datasets and three-dimensional surface contours, providing detailed characterization of the welding interface. Subsequently, advanced big data analytics integrated with machine learning algorithms are employed to evaluate and predict welding quality parameters with enhanced accuracy. Furthermore, machine learning techniques offer innovative solutions for optimizing the mesh element distribution in finite element modeling of Cu/Al ultrasonic welding processes, thereby improving the simulation fidelity and predictive capability of the numerical models.
